# Metabolomics analyses to characterize metabolic alterations in Korean native calves by oral vitamin A supplementation

**DOI:** 10.1038/s41598-020-65023-y

**Published:** 2020-05-15

**Authors:** Dong Qiao Peng, Seong Jin Kim, Hong Gu Lee

**Affiliations:** 1 Department of Animal Science and Technology, Sanghuh College of Life Sciences, Konkuk University, Seoul, 05029 Korea; 2 Team of an Educational Program for Specialists in Global Animal Science, Brain Korea 21 Plus, Sanghuh College of Life Sciences, Konkuk University, Seoul, 05029 Korea; 3 Asia Pacific Ruminant Institute, Icheon, 467814 Korea

**Keywords:** Metabolomics, Animal physiology

## Abstract

Previous studies have reported that vitamin A administration in the birth stage of calves could promote preadipocyte and muscle development. However, the metabolic change after vitamin A administration remains unknown. Thus, the objective of this study was to perform metabonomics analyses to investigate the effect of vitamin A in Korean native calves. Ten newborn calves (initial average body weight: 30.4 kg [SD 2.20]) were randomly divided into two groups treated with or without vitamin A supplementation (0 IU vs. 25,000 IU vitamin A/day) for two months until weaning. Metabolic changes in the serum and longissimus dorsi muscle of calves were investigated using GC-TOF-MS and multivariate statistical analysis. As a result, ten metabolic parameters in the serum and seven metabolic parameters in the longissimus dorsi muscle were down-regulated in the vitamin A treatment group compared to those in the control group (VIP value > 1.0, *p* < 0.05). Both serum and longissimus dorsi muscle showed lower levels of cholesterol and myo-inositol in the vitamin A treatment group than in the control group (*p* < 0.05). These results indicate that vitamin A supplementation in the early growth period of calf could maintain the preadipocyte status, which can contribute to future adipogenesis in the intramuscular fat production of Korean native cattle.

## Introduction

To satisfy the demand of consumers and provide a profit to producers, beef with a high percentage of intramuscular fat (marbling score) is highly demanded in beef production industry. The negative relationship between serum vitamin A and beef marbling score during the fattening stage was firstly observed in Japanese Black steers^1^. After that, numerous studies have been conducted using vitamin A restriction strategy to increase the marbling score of different species of cattle/steers^2–4^. Under vitamin A restriction during the fattening stage, some metabolic biomarkers in different species of cattle/steers were found, including higher levels of serum glucose, urea nitrogen, albumin, and magnesium in Japanese Black steers^5^, and the increasing level of albumin, urea nitrogen, creatinine, and non-esterified fatty acid in Korean native steers^6^.

Recently, Wang, *et al*.^7^ have hypothesized that vitamin A supplementation from late pregnancy of cattle to postnatal of the calf could also increase the development of intramuscular fat by adipocyte hyperplasia. In an *in vivo* trial, a high concentration of vitamin A was injected into the calf until reaching two months age. As a result, the vitamin A treated group showed a higher level of Zinc finger protein 423 (Zfp423) gene expression level, which related to preadipocyte development, as well as increase the marbling score in the final carcass traits^8^. In addition, another study showed that vitamin A treatment promoted muscle development by inducing myogenic markers and increased the oxidative muscle fibers in two months old calves^9^. Moreover, our previous study has shown that vitamin A supplementation from late pregnancy to two months old calf could increase postnatal body weight and expression of genes related to preadipocyte development and muscle development (unpublished). However, metabolic changes related to preadipocyte and muscle development during postnatal stage in calves still remain unclear. Therefore, the objective of this study was to investigate metabolic changes related to preadipocyte and muscle development in calves under vitamin A supplementation in postnatal two months old.

## Materials and methods

### Animal and management

All experimental procedures and processes were conducted in accordance with the “Guidelines for Care and Use of Experimental Animals” of Konkuk University (Approval no: KU17099). Twenty-seven Korean native (Hanwoo) heifers were selected (2^nd^ parity; initial average BW = 320 kg [SD 31.2]) as candidate subjects in this research. All heifers were randomly placed in multiple pens with a stainless steel gate and automatic water fountain. Clean, dry wood dust was spread on the floor for bedding, which was changed every month. Heifers were pregnant (AI method) using semen (non sexed) from different Korean native cattle. The length of pregnancy was expected to be 280 days. Diets were fed to pregnant heifer at 0900 h in the morning, and the quantity and nutrient contents of feedstuff were determined according to the Korean feeding standard for Hanwoo^10^ (Table S1). All newborn calves (male: 19 heads; female: 8 heads) from candidate heifers were weighed at birth and immediately separated from their dams to individual wooden pens (200 cm (L) × 135 99 cm (B) × 120 cm (H)). Ten male calves were selected for this study based on the similar body weight and the similar date of birth (initial average body weight: 30.4 kg [SD 2.20]), then these calves were randomly assigned to the control and the treatment group. This was followed by feeding 3.6 L of commercial colostrum (225 g in 625 mL of 45 °C water, HEADSTART, NAC No. 15360020, Saskatoon Colostrum Company Ltd., Saskatoon, SK, CA) at 12 hours after birth. Buckets for clean water and feedstuff were prepared for each calf, as well as a commercial milk replacer (NUKAMEL YELLOW, The Dawn Ltd., Seoul, Republic of Korea). For diet, the milk replacer (dissolved 140 g in 1 L, 45 °C water) was fed to each calf according to an automatic calf feeder (FOERSTER TECHNIK, Gerwigstraße 25, 78234 Engen, Germany) six times a day with a maximum volume setting of 6.1 L (at approximately 20% of birth weight) until one-month age, followed by slow gradient descent until two-months age. Meanwhile, feedstuff mixed with 80% commercial concentrate (DH VITAL FEED, Gyeonggi-do, Republic of Korea) and 20% forage (alfalfa) was fed to calves, which guided calves to ingest within one-month age. The feeding amount was increased from one-month to two-month of age, with the reduction of milk replacer supply. In addition, clean water and feedstuff were fed *ad libitum*. Dry sawdust was changed every week during the experimental period. Moreover, the health status of the calves was checked every day. Those who presented clinical diarrhea were injected with biodyl, Baytril, ketoprofen, and oral nutritional supplement formula (LIFE-AID, Norbrook, Northamptonshire, UK) mixed with Neodiaristin, which was administered to calves at the same time. In addition, Ampura-q administration was conducted in one-month age calf to prevent coccidiosis. The total feed intake (g/day of DM) was recorded daily during the experimental period, however there was no significant difference between control and the treatment group. The basal vitamin A intake in the control and treatment group was approximately 20,000 IU/ per day (calculated from Table S1). Additional vitamin A supplementation in treatment group (25,000/IU per day, retinyl acetate, HANYOU FEED, Wuxi, China) was administered with the milk replacer from day 5 of birth to two-month of age.

### Blood sample collection

All blood samples were obtained from the jugular vein of each individual (10 ml) with 18GX needles at 0900 h when the calves were two-month of age. Blood for metabolomics analysis was prepared with a clot activator tube (BD VACUTAINER, Belliver Industrial Estate, Plymouth, UK). After blood sampling, all containers were transported to the lab by an opaque box with ice bags. The whole blood was then centrifuged at 3,500 rpm (radius: 155 mm) for 15 min at 4 °C after standing at room temperature (RT) for one hour. Then the serum for metabolomics analysis was separated into 1.5 mL micro-tubes and then stored at −80 °C for future analysis.

### Longissimus muscle collection

Longissimus muscle samples (12th to 13th ribs, approximately 2 g) were collected by surgery biopsy from five heads of male calves in each group when the calves were two-month of age. All the biopsy procedure was operated by the veterinarian in accordance with the “Guidelines for the Care and Use of Experimental Animals” of Konkuk University. The sampling procedure were conducted at the same day of blood sampling but after taking blood at 1400. After biopsy procedure, the management was conducted after the surgery up to two weeks according to the veterinarian’s prescription. All obtained samples were immediately put into 5 mL microtubes, quick-frozen in liquid nitrogen, then transported into a box full of dry ice until arrival at the laboratory. All muscle samples were stored in −80 °C deep freezer before use for the experiment.

### GC-TOF-MS analysis for serum and longissimus muscle

Pretreatment and GC-TOF-MS process was conducted according to a previous study^11^. Briefly, for serum and longissimus samples, they were thawed at 4 °C. After 1 mL of 100% methanol was added to each sample, samples were shaking with a mixermill at a frequency of 30 s^−1^ for 10 min. After that, samples were placed in a refrigerator room (−20 °C) to cool down for one hour then centrifugated at 13,000 rpm for 10 min at 4 °C. The supernatant was filtered by a 0.2 μm polytetrafluoroethylene filter and dried by speed vacuum (MODULSPIN 31; biotron,Korea). Dried samples were prepared for further GC-TOF-MS analysis.

Oximation and silylation processes were conducted for GC-TOF-MS analysis. Dried samples were dissolved in 50 μL of methoxyamine hydrochloride (methyloxime derivative) for 90 min at 30 °C. These samples were then silylated by adding 50 μL MSTFA and incubated at 37 °C for 30 min. After silylation, GC-TOF-MS analysis was conducted using an Agilent 7890 A gas chromatography system (AGILENT TECHNOLOGIES, Palo Alto, CA, USA) equipped with an Agilent 7693 autosampler (AGILENT TECHNOLOGIES) and a PEGASUS HT-TOF-MS (LECO, St. Joseph, MI, USA) system. Samples were separated with a Rtx-5MS column (i.d. 30 m × 0.25 mm × 0.25 μm, Restek Corp., Bellefonte, PA, USA) with a flow of 1.5 mL/min helium as the carrier gas. A microliter aliquot of sample was injected into the GC with no split. For the analysis, the initial oven temperature was held at 70 °C for 2 min. The temperature was then raised 15 °C per min until it reached 300 °C. The temperature was then held at 300 °C for 3 min. Temperatures of front inlet and transfer line were 250 °C and 240 °C, respectively. Electron impact ionization (70 eV) was utilized. Mass data were obtained at the range of 50–1000 m/z. Each sample was conducted for two analytical replications.

### Data preprocessing and statistical analysis

Data preprocessing and statistical analysis were performed according to a previous study^11^. Raw data collected from GC-TOF-MS were converted into CDF (NetCDF) files with a LECO CHROMA TOF software (version 4.44; LECO Corp.). Peak detection, retention time correction, and alignment were then processed with an online METALIGN software package (http://www.metalign.nl). After that, final data were transformed into Excel files. Data were then arranged on a three-dimensional matrix, including information on the peak, peak area, and sample name. Multivariate statistical analysis was performed with SIMCA-P^+^ (version 12.0, Umetrics; Umea, Sweden). Unsupervised principal component analysis (PCA) was conducted to investigate the general aggregate state and trends in different groups among all samples. A model of supervised orthogonal partial least squares discriminant analysis (OPLS-DA) was performed to maximize metabolic change and determine significantly alternated metabolites in different groups. Variables were separated based on variable importance in the projection (VIP) value. Finally, the statistical difference between control and treatment groups was assessed using independent (unpaired) samples t-tests for unequal variances in SAS 9.4 (SAS Institute Inc., Cary, NC, USA). Differences observed between means were considered significant at *p* < 0.05.

## Results

### Metabolic profiles of control and vitamin A treatment group

**Figure 1:**
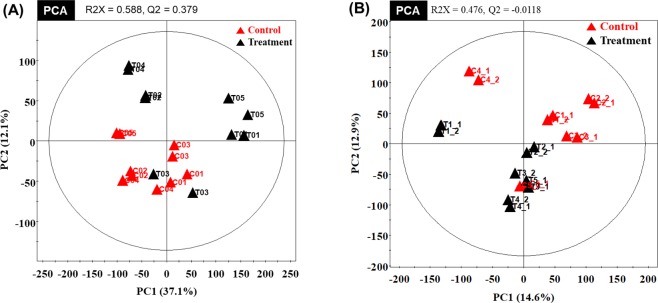
PCA (principal component analysis) score plot of the control group (▲ red triangles) and the treatment group (▲ black triangles) in serum and longissimus muscle. (**A**) PCA analysis of serum. (**B**) PCA analysis of longissimus muscle.

**Figure 2:**
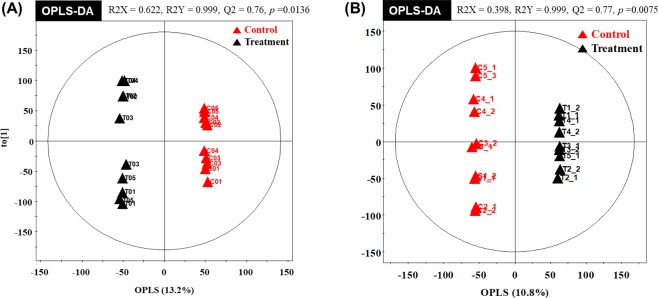
OPLS-DA (partial least squares discriminant analysis) score plot and isolated metabolic parameters (VIP > 1.0) of the control group (▲ red triangles) and the treatment group (▲ black triangles) in serum and longissimus muscle. (**A**) OPLS-DA analysis of serum. (**B**) OPLS-DA analysis of longissimus muscle.

Metabolic raw data of serum and longissimus muscle were collected from a multivariate statistical analysis. The status of metabolites in control (**▲**red triangles) or vitamin A treatment group (**▲**black triangles) was determined in the score plot for principal component analysis (PCA) from serum and longissimus muscle, respectively (Fig. 1). The distribution of metabolite parameters in serum (R^2^X = 0.622, R^2^Y = 0.999, Q^2^ = 0.76, *p* = 0.0136) and longissimus muscle (R^2^X = 0.398, R^2^Y = 0.999, Q^2^ = 0.77, *p* = 0.0075) showed a clear separation in the OPLS-DA model (Fig. 2).

### Detection and identification of metabolic markers

**Figure 3:**
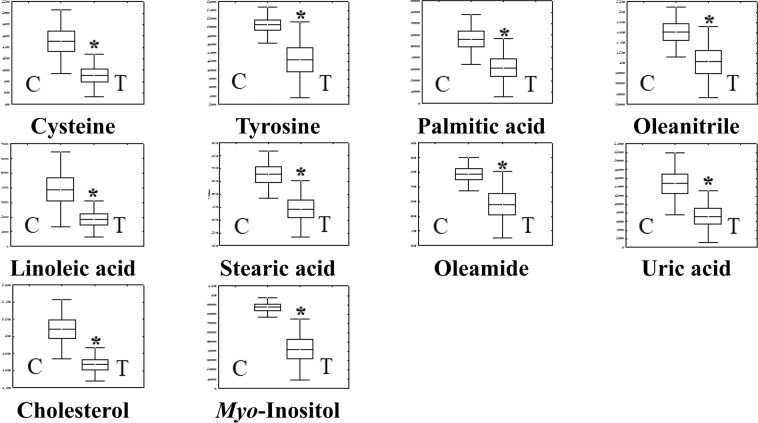
Metabonomics parameters (n = 10) in Serum (Con vs. vitamin A treatment; VIP > 1.0, *p* < 0.05). Values (mean ± SEM) with asterisk (*) differ significantly compared to the control group (*p* < 0.05).

**Figure 4:**
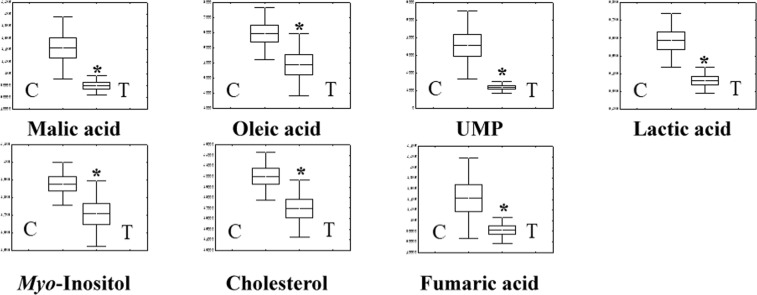
Metabonomics parameters (n = 7) in longissimus muscle (Con vs. vitamin A treatment; VIP > 1.0, *p* < 0.05). Values (mean ± SEM) with asterisk (*) differ significantly compared to the control group (*p* < 0.05).

Detection and identification of metabolic markers were based on results from databases of HMDB (http://www.hmdb.ca/), PUBCHEM (https://pubchem.ncbi.nlm.nih.gov/) and KEGG (http://www.genome.jp/kegg/). According to the OPLS-DA analysis, potential metabolic markers were separated from the serum and longissimus muscle based on the value of importance in the projection higher than 1.0 (VIP > 1.0). As results from serum samples, cysteine, tyrosine, palmitic acid, oleanitrile, linoleic acid, stearic acid, oleamide, cholesterol, uric acid, and myo-inositol in the vitamin A treatment group (ten metabolic parameters) displayed significantly (*p* < 0.05) lower levels than those in the control group (Fig. 3). From longissimus muscles, seven metabolic parameters (oleic acid, cholesterol, lactic acid, malic acid, fumaric acid, myo-inositol, and uridine 5′-monophosphate (UMP)) were found to be decreased significantly (*p* < 0.05) in the vitamin A treatment group (Fig. 4). Interestingly, we found that cholesterol and myo-inositol were both decreased in serum and longissimus muscle (Fig. 7).

**Figure 5:**
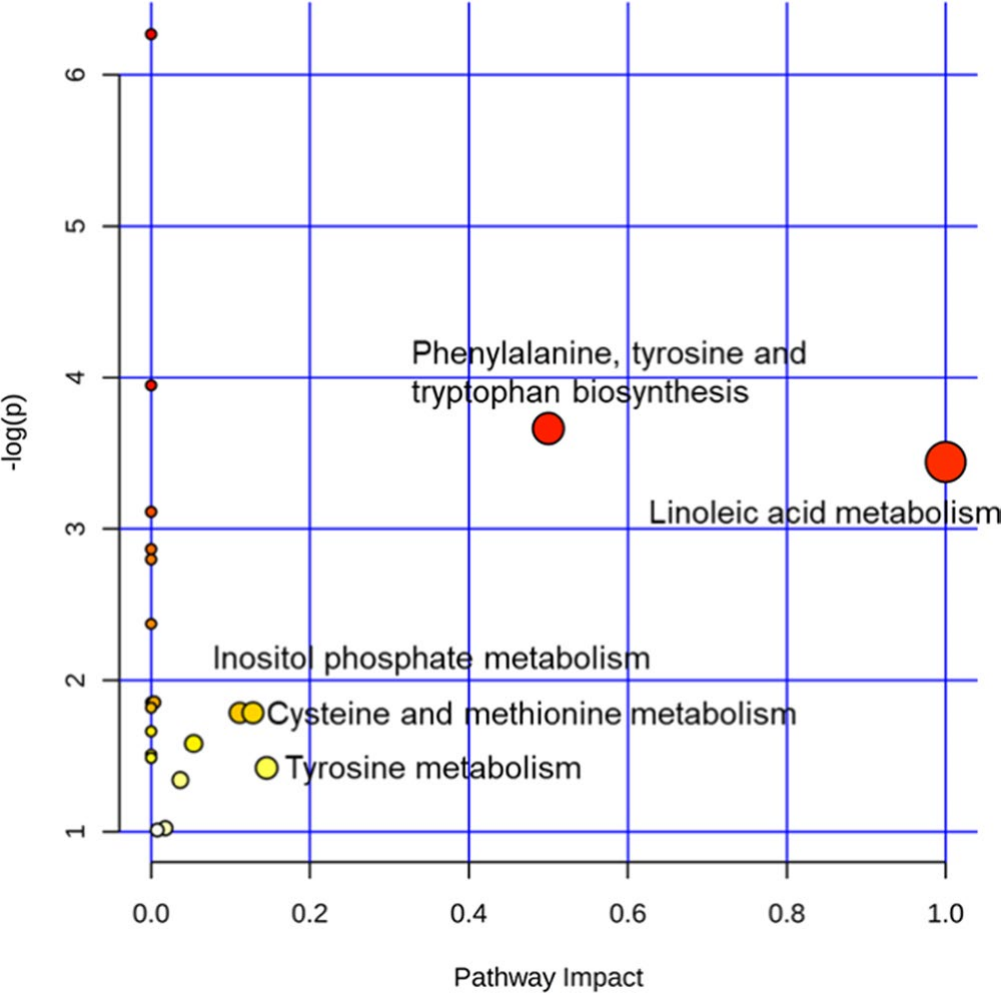
The metabolome map of relevant metabolic pathways for change in control and vitamin A treatment groups of serum metabolic profiles.

**Figure 6:**
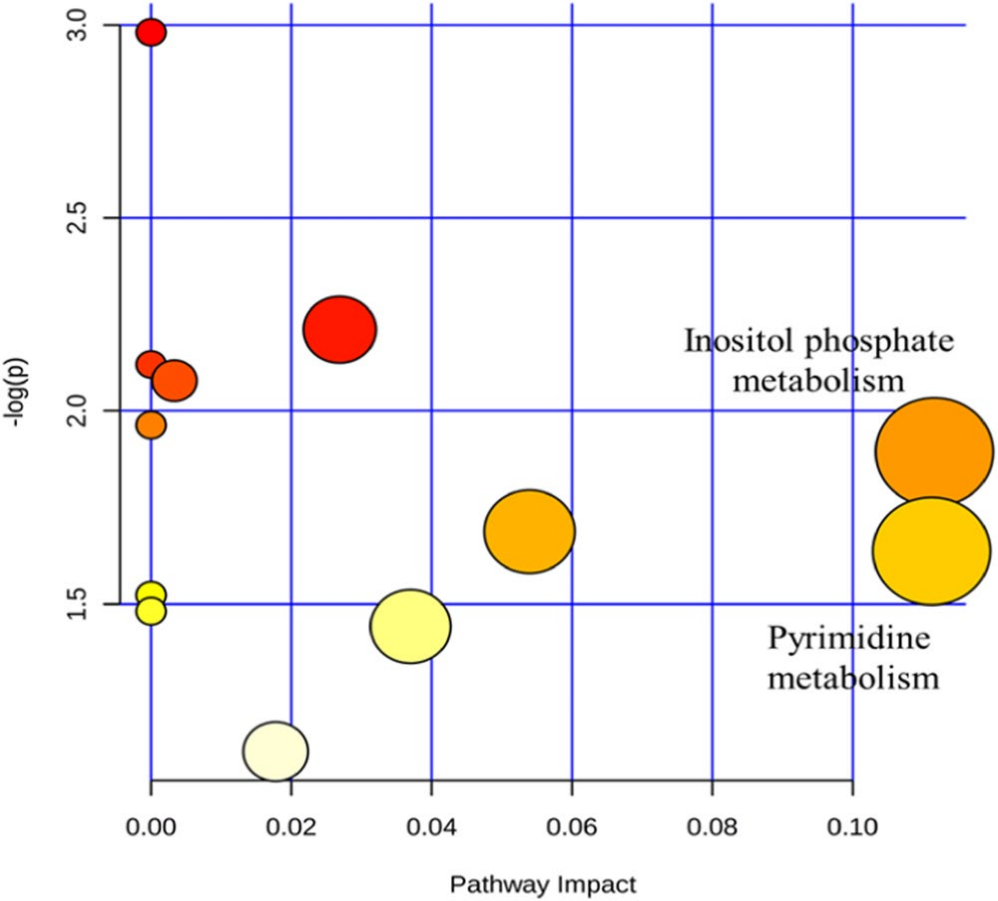
Metabolome map of relevant metabolic pathways for change in control and vitamin A treatment groups of longissimus muscle metabolic profiles.

**Figure 7:**
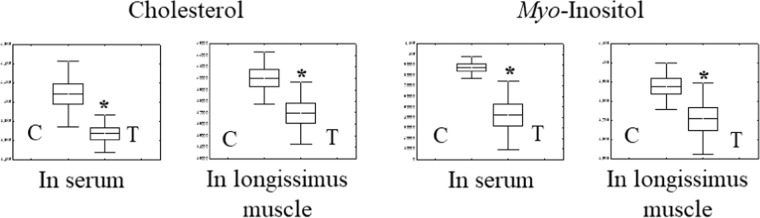
Both cholesterol and *Myo*-Inositol levels are reduced in serum and longissimus muscle of Korean native calves under vitamin A supplementation. Values (mean ± SEM) with asterisk (*) differ significantly compared to the control group (*p* < 0.05).

### Characterization and functional analysis of metabolic pathways in serum and longissimus muscle

Metabolic pathways were defined with an online MetPa system (METABOANALYST 4.0, http://www.metaboanalyst.ca/). Metabolites with significant changes in serum and longissimus muscle were imported into the online metaboanalyst system to generate the metabolome view list. In the model organism interface, *Bos taurus* was selected for pathway analysis. An over-representation analysis was utilized for the pathway enrichment analysis. The pathway topology analysis was based on the relative betweenness centrality measure in the established metabolic network to predict the importance of metabolites. Potential targets were selected based on both impact value (not below 0.1) and *p*-value (no more than 0.05). Figures 5 and 6 show metabolome maps of relevant metabolic pathways in serum and longissimus muscle based on the KEGG database, respectively^12–14^. Potential function pathways were summarized in Table 1. Vitamin A treatment resulted in significant changes of phenylalanine-tyrosine and tryptophan biosynthesis (*p* = 0.026, impact = 0.50), linoleic acid metabolism (*p* = 0.032, impact = 1.00) in the metabolic pathway for the serum. However, no significant change was found in inositol phosphate metabolism (*p* = 0.168, impact = 0.11), cysteine or methionine metabolism (*p* = 0.168, impact = 0.13), and tyrosine metabolism (*p* = 0.242, impact = 0.15). As for the longissimus muscle, vitamin A treatment only resulted in changes in inositol phosphate metabolism (*p* = 0.151, impact = 0.11) and pyrimidine metabolism (*p* = 0.194, impact = 0.11), without reaching statistical significance. From our previous results (unpublished data), we found that vitamin A treatment in calves had a prominent influence on preadipocyte and muscle development. However, we did not find the pathway related to our new findings in the serum and longissimus muscle from metabolic analysis.

**Table 1:** Detailed results of potential metabolic pathways for control and vitamin A treatment groups.

Metabolite pathway	p-value	Impact	Metabolites
**Serum**			
Phenylalanine, tyrosine and tryptophan biosynthesis	0.026	0.50	L-Tyrosine
Linoleic acid metabolism	0.032	1.00	Linoleic acid
Inositol phosphate metabolism	0.168	0.11	Myoinositol
Cysteine and methionine metabolism	0.168	0.13	Cysteine
Tyrosine metabolism	0.242	0.15	L-Tyrosine
**Longissimus muscle**			
Inositol phosphate metabolism	0.151	0.11	Myo-Inositol
Pyrimidine metabolism	0.194	0.11	UMP

UMP: Uridine 5′-monophosphate.

## Discussion

In a previous study, metabolic changes were investigated during vitamin A restriction in the cattle. Results showed that serum levels of glucose, urea nitrogen, albumin, and magnesium were elevated in Japanese Black steers^5^. Serum albumin, urea nitrogen, creatinine, and non-esterified fatty acid in Korean native steers were also found to be increased by vitamin A restriction^15^. Alternated metabolic parameters suggest a potential index to indicate changes under vitamin A restriction during the fattening period in different species of cattle/steers. Recently, vitamin A supplementation in the early growth period of cattle has resulted in a higher marbling score in future carcass trait evaluation^8^. However, metabolite changes in the control and vitamin A treatment group remained unknown.

In this study, the basal vitamin A intake in the farm was approximately 20,000 IU/day of vitamin A to each calf, which was nearly two times of the recommended amount for calves in NRC (2001) (recommended vitamin A concentration: 9,000 IU/kg of DM) experimental period^16^. In order to investigate the effect of vitamin A, we decided to feed extra 25,000 IU/day of vitamin A to the calves; therefore totally, 45,000 IU/day of vitamin A was fed to the calves at approximately five times of the recommended amount^16^, and 2 times higher than the current farm procedure in the treatment group. A previous study^17^ reported that the milk replacer with supplemental vitamin A at 44,000 IU/kg of DM caused an increased vitamin A deposition in the liver, without showing symptoms of toxicity in Holstein calves. Furthermore, ruminants exhibited a higher vitamin A tolerance, which may due to the degradation function by rumen microbes^18^. In line with this result, a precedent study showed that vitamin A supplementation up to 132,000 IU per 100 kg of BW per day caused tissue damage in male calves^19^.

In the current study, phenylalanine, tyrosine, and tryptophan biosynthesis, and linoleic acid metabolism were found to be downregulated after vitamin A supplementation according to serum metabolite pathway analysis. Phenylalanine, tyrosine, and tryptophan are assigned to aromatic amino acids (AAA) in the synthesis of protein. The most recent review has summarized the importance of the metabolism of AAA for the health of the host animal and its resident microflora^20^. They demosntarted that the low level of AAA in vitamin A treatment group indicated the catabolism of phenylalanine, tyrosine, and tryptophan. They also reported that AAA taken by animals could be broken down and then transformed into other crucial material for the development of the animal. For example, phenylalanine could be transformed into tyrosine for the biosynthesis of neurotransmitter. Tryptophan can be considered as a precursor for the production of serotonin, tryptamine, neurohormone melatonin, and vitamin niacin for the synthesis of neurotransmitters^20,21^. In the current study, vitamin A treatment resulted in downregulation of linoleic acid metabolism via serum samples. However, for the metabolite set in the longissimus muscle, we could not find any change in linoleic acid. Linoleic acid (LA) is a polyunsaturated omega-6 fatty acid that affects fat metabolism and health of the animal. A previous study on mice showed that offspring from wild-type mice mothers supplied with an LA had higher fat enrichment than the LA/alpha-linolenic acid fed ones, indicating that the balance of necessary polyunsaturated fatty acids might adjust the development of adipose tissue in the early growth stage of animal^22^. However, linoleic acid (CLA) can also reduce adipogenic differentiation in 3T3-L1 cell line^23^. A metabolic syndrome rat model has also demonstrated that fat hypertrophy is related to the decrease of the linoleic acid in intra-abdominal adipocyte^24^. In addition, a recent review has stated a perspective that LA synthesis is related to a phylogenetic framework in the animal under different selective pressures^25^. These results indicate that vitamin A treatment not only plays an essential act in adipocyte development but also plays a vital role in several aspects of animal which needs further investigations.

Interestingly, in the current study, we found that cholesterol and myo-inositol were significantly decreased in the serum and longissimus muscle after vitamin A treatment. Cholesterol contributes to triglyceride accumulation in the process of adipocyte hypertrophy. It also plays an pivotal role in the early phase and late phase of adipocyte formation^26,27^. Myo-inositol is one of compounds of inositol. It is chemically identified as hexahydroxycyclohexane with nine stereoisomers^28^. Myo-inositol can increase adipogenesis and improve glucose uptake in 3T3-L1 adipocytes^29^. It is also involved in the insulin signaling regulation and glucose uptake^28^. Thus, according to the previous studies, both cholesterol and myo-inositol play a crucial role in adipogenic differentiation in the process of adipocyte hypertrophy. However, our result showed the lower level of cholesterol and myo-inositol in the vitamin A treatment group than in the control group, indicating that stimulation of adipogenic differentiation process was depressed in the vitamin A treatment group. Conversely, the preadipocyte or the status of adipocyte precursors could be considered to be maintained under vitamin A treatment situations. Taken together, these results suggest that vitamin A treatment during the early growth period of calves may contribute to the development of preadipocyte or adipocyte precursors rather than terminal adipogenic differentiation.

## Conclusion

In the current study, vitamin A supplementation was administrated to calves from birth to two-month of age. Our results revealed that a two times amount of vitamin A supplementation (25,000 IU/day) downregulated phenylalanine, tyrosine, and tryptophan biosynthesis and linoleic acid metabolism in the serum. In addition, in the vitamin A treatment group, cholesterol, and myo-inositol were decreased in both serum and longissimus muscle. These results indicated that vitamin A supplementation in the early growth stage of the calf had the potential to maintain preadipocyte or adipocyte precursor cells development, rather than terminal adipogenic differentiation. Finally, the change of these bio-markers may lay a theoretical foundation for future research on preadipocyte development and the health of calf.

## Supplementary information

Supplementary information.

## Data Availability

Datasets generated during and/or analyzed during the current study are available from the corresponding author upon reasonable request.
