# Supplementary material for: Metabolomics analyses to characterize metabolic alterations in Korean native calves by oral vitamin A supplementation
Source: Sci Rep. 2020 May 15;10:8092. doi: 10.1038/s41598-020-65023-y (PMC7228984; doi:10.1038/s41598-020-65023-y)
Supplement: Supplementary file 1 — Supplementary information. [file 41598_2020_65023_MOESM1_ESM.docx]

**Table S1. Basic nutrient composition of the experimental diets in pregnant cattle and calf.**

| Component | Pregnant cattle |  | Calf | | |
| --- | --- | --- | --- | --- | --- |
|  | Total mixed ration |  | Milk replacer powder | Roughage | Concentrate |
| DM (%) | 44.02 |  | 94.70 | 90.19 | 88.92 |
| Crude protein (% DM) | 10.26 |  | 24.14 | 15.54 | 25.93 |
| Crude fat (% DM) | 3.70 |  | 16.68 | 1.71 | 6.67 |
| Crude fibre (% DM) | 26.58 |  | 0.33 | 34.73 | 8.21 |
| Crude ash (% DM) | 14.42 |  | 7.66 | 9.39 | 6.68 |
| ADF^1^ (% DM) | 34.76 |  | - | 40.77 | 10.59 |
| NDF^2^ (% DM) | 57.91 |  | - | 53.51 | 28.61 |
| Calcium (% DM) | 0.77 |  | 0.81 | 1.39 | 0.87 |
| Phosphorus (% DM) | 0.32 |  | 0.64 | 0.26 | 0.56 |
| GE^3^ (kcal/g of DM) | 4.21 |  | 5.08 | 4.17 | 4.64 |
| Vitamin A (IU/ kg) | 100,000 |  | 25,000 | 7,860 | 20,000 |

^1^ADF: Acid detergent fiber

^2^NDF: Neutral detergent fiber

^3^GE: Gross energy (analyzed in laboratory)

**Title:** Metabolomics analyses to characterize metabolic alterations in Korean native calves by oral vitamin A supplementation

**Author details**

Dong Qiao Peng:

^1^Department of Animal Science and Technology, Konkuk University, Seoul 05029, Republic of Korea

^2^Team of an Educational Program for Specialists in Global Animal Science, Brain Korea 21 Plus Project, Konkuk University, Seoul 05029, Republic of Korea

Seong Jin Kim:

^3^Asia Pacific Ruminant Institute, Icheon 467814, Republic of Korea

Hong Gu Lee:

^1^Department of Animal Science and Technology, Konkuk University, Seoul 05029, Republic of Korea

^2^Team of an Educational Program for Specialists in Global Animal Science, Brain Korea 21 Plus Project, Konkuk University, Seoul 05029, Republic of Korea

^*^Corresponding author, Tel: +82-2-250-0523, Fax: +82-2-255-1044, E-mail: hglee66@konkuk.ac.kr
